# Outcomes of Descemet Membrane Endothelial Keratoplasty (DMEK) Using Surgeon’s Prepared Donor DM-Roll in Consecutive 100 Indian Eyes

**DOI:** 10.2174/1874364101812010134

**Published:** 2018-07-23

**Authors:** Samar K Basak, Soham Basak, Viraj R Pradhan

**Affiliations:** Cornea Department, Disha Eye Hospitals, Kolkata - 700120, India

**Keywords:** Visual Outcomes, Descemet Membrane Endothelial Keratoplasty, Surgeon’s prepared tissue, MK medium preserved-cornea, Cornisol-preserved cornea, Indian Eyes

## Abstract

**Background:**

Descemet Membrane Endothelial Keratoplasty (DMEK) is now becoming the popular form of endothelial keratoplasty using only donor DM with healthy endothelium as true component lamellar corneal surgery.

**Objective:**

To analyze the results of visual outcomes, endothelial cell loss and complications of Descemet membrane endothelial keratoplasty in first consecutive 100 Indian eyes.

**Methods:**

100 eyes of 95 consecutive patients with endothelial dysfunctions of different etiologies scheduled for DMEK, were included in this study. In each case, surgeon prepared tissue using McCarey Kaufman medium- or Cornisol-preserved donor cornea with a cell count of ≥2500 cells/mm^2^. Surgical complications, Best Spectacle Corrected Visual Acuity (BSCVA); Endothelial Cell Density (ECD) and Endothelial Cell Loss (ECL) were analyzed for each patient after a minimum follow-up of three months.

**Results:**

The Main indication was pseudophakic corneal edema or bullous keratopathy in 52 (52%) eyes. 38 (38%) eyes had Fuchs′ dystrophy with various grades of cataract. In 43 phakic eyes, DMEK was combined with cataract surgery and intraocular lens implantation. Mean DM-roll preparation time was 7.5 ± 2.8 min and in 3 eyes, DM-graft were damaged. After 3-months, BSCVA was ≥20/25 in 57 (57.6%) cases. Mean ECD was 2123 ± 438/mm^2^ (range: 976 - 3208/ mm^2^) and the mean endothelial cell loss after 3-months was 26.92 ± 13.40 (range: 4.90 - 66.6%). Partial DM detachment occurred in 8 (8.0%) eyes and rebubbling required in 4 eyes. Iatrogenic primary graft failure occurred in one eye.

**Conclusion:**

Descemet membrane endothelial keratoplasty is a safe and effective procedure in several types of endothelial diseases among Indian patients with encouraging surgical and visual outcomes. Complications are less and endothelial cell loss percentage is acceptable.

## 1 INTRODUCTION

Endothelial Keratoplasty (EK) is now well established as the treatment of choice for corneal endothelial diseases, like Pseudophakic Corneal Edema (PCE) or Pseudophakic Bullous Keratopathy (PBK) and Fuchs’ Endothelial Corneal Dystrophy (FECD) [1]. Compared to PK, it offers the advantages of faster visual rehabilitation, better refractive outcomes, better tectonic support and lesser chances of infection or rejection [2]. Descemet stripping (automated) endothelial keratoplasty (DSEK/DSAEK) is the most common type of EK procedure across the world. However, DSAEK is not a true anatomic replacement surgery. The stroma-to-stroma interface irregularities and a slight hyperopic shift which causes little delayed and less than perfect visual outcomes [3].

In 2006, Melles *et al*. performed a pure Descemet's Membrane (DM)-endothelium complex transplant which is a true anatomical replacement surgery and named it Descemet Membrane Endothelial Keratoplasty (DMEK) [4]. This procedure has shown potentially better and faster visual outcomes than DSAEK, though the initial learning curve is much steeper and there is more Endothelial Cell Loss (ECL) due to more donor manipulation during surgery. However, with time, DMEK has been evolved as a standardized, “no-touch” procedure, with better results in terms of ECL [5, 6]. In fact, DSAEK surgery in the USA, is gradually but slowly decreasing in numbers in each of the past three years, while DMEK procedure has increased by 64% in 2015 and by 37.6% in 2016 [7].

Unlike Western patients, Indian patients present late in the clinic and most of the eyes with best spectacle corrected visual acuity of <20/200 [8]. Moreover, in presence of deep brown eyes DMEK donor unscrolling becomes difficult. Indian Eye Banks also do not supply pre-peel DM-roll to the surgeon and most of the eye banks preserve cornea either in McCarey Kaufman Medium (MKM) (RIEB, LV Prasad Eye Hospital, Hyderabad, India) or in Cornisol medium (CSM) (AuroLab, Madurai, India) [9]. Till date, there is only study from India with 40 eyes where the surgeons used CSM-preserved donor cornea. In that study, they concluded that DMEK is a feasible procedure for endothelial pathologies in presence of shallow anterior chamber in Asian eyes [10].

The purpose of the present study was to evaluate the surgical and visual outcomes, Endothelial Cell Loss (ECL) and complications following DMEK surgery by surgeon’s prepared donor graft among the Indian population.

## 2 MATERIALS AND METHODS

It was a prospective, non-randomized, non-comparative interventional surgical case series. The study was approved by the institutional review board, and a special informed written consent was taken from all patients prior to surgery. The first 100 consecutive eyes of 95 patients with endothelial diseases of various etiologies were included for DMEK procedure.

Preoperatively all patients underwent the Best Spectacle Corrected Visual Acuity (BSCVA) using the Snellen chart, slit lamp examination, Goldmann’s Applanation Tonometry (GAT) and dilated fundus examination with direct and/or indirect ophthalmoscope if possible. Intraocular pressure (IOP) was checked with Noncontact Tonometer (NCT) in eyes where GAT was not possible. Ultrasonography (USG) B- scan was done in some of the eyes where the fundus details were not clearly visible.

The *exclusion criteria* were gross corneal edema with poor visibility, corneal stromal scarring, irregular and deformed Anterior Chamber (AC), vitreous in AC, extensive Peripheral Anterior Synechia (PAS), uncontrolled glaucoma, and gross posterior segment pathology as detected by USG B-scan. In all phakic eyes, DMEK was combined with cataract surgery, performed by phacoemulsification with implantation of hydrophobic acrylic posterior chamber intraocular lens (PE-PCIOL); the DMEK-triple. All surgeries were performed by a single surgeon (SKB) in the same tertiary care setting.

### 2.1 Donor Tissue and DM-roll Preparation

DMEK donor roll was prepared first in the operation room by the surgeon just prior to DMEK procedure. Healthy Corneo-Scleral (CS) buttons, preserved in MKM or CSM were supplied by the eye bank situated within the hospital. The selected donor age was between 40 and 80 years with an Endothelial Cell Density (ECD) of ≥2500 cells/mm^2^ as measured by eye bank specular microscope (KeratoAnalyzer – EKA-10, Konan Medical Inc., Hyogo, Japan). CS button was first placed on a Teflon block and 9.5 mm partial trephination was done. Peripheral DM beyond the trephination mark was stripped first. Then it was peeled off from the posterior stroma with the help of McPhearson’s forceps. A paracentral full thickness 3 mm trephination was done through the stroma. DM was floated back and placed in its original position. Whole CS button was flipped and ‘S’ stamp was put on the DM side after drying. The hole was covered and again flipped back with the endothelial side up. A 7.5 to 8.25 partial trephination of the cornea was made. This DM-endothelial complex, *i.e.,* the donor graft was completely separated with the help of McPhearson’s forceps and then stained with Trypan Blue for 30 to 45 seconds. DM-roll or scroll was formed spontaneously due - to its elastic property, with the endothelial layer on its outer side. Stained DM-roll was then temporarily transferred to a small petri dish, containing a Balanced Salt Solution (BSS) and ready for suction into an injector system.

### 2.2 Recipient Eye Preparation and Surgical Technique

All surgeries were performed under conventional peribulbar or sub-Tenon anesthesia. In pseudophakic eyes, the pupil was constricted by instillation of three drops of 2% pilocarpine eye drops 30 min prior to surgery. Pupillary dilation was required in all phakic eyes where it was combined with PE-PCIOL.

- In recipient eye, 8.5 mm diameter template mark with gentian violet was made on the epithelial surface which served as a reference mark for Descemet stripping. In some eyes, the loose edematous epithelium was removed before marking.
- A 3-mm tunnel incision was made superiorly at the posterior limbus with an angular keratome, entering the Anterior Chamber (AC).
- Two 1-mm side-port incisions were made on either side of the main incision at 10 and 2 o′clock positions. These were to manipulate the DM-roll with BSS and intermittent decompression of AC and to inject air.
- Cohesive viscoelastic agent, 1% Sodium hyaluronate (Healon, Advanced Medical Optics, Inc, Uppsala, Sweden) was injected into the AC.
- In patients who underwent a DMEK-triple, at this step, PE-PCIOL surgery was performed by the same surgeon using the same tunnel and side ports. Every attempt was made to overlap the capsulorhexis margin by 1 mm on the optic of the IOL. The IOL power was calculated from the biometry of the same or the other eye, or from the spectacle history, and to aim 0.5 D myopia as there is a little/no hyperopic shift in DMEK [11, 12]. The pupil was then constricted with intracameral pilocarpine (0.5%) injection.
- Circular dissection of the Descemet membrane (Descemetorhexis or Descemet scoring) was carried out with a reverse Sinsky′s hook which corresponded to the 8.5 mm epithelial template mark. DM was completely stripped off with the help of the hook, and the diseased tissue was removed. In some cases, with severe corneal edema with poor visibility, trypan blue (0.06%) solution was used to stain the diseased endothelium for better visibility.
- An inferior Peripheral Iridotomy (PI) was performed with the help of a vitrector. A thorough AC wash was given with BSS followed by temporary air tamponade to stop oozing of blood from PI site. After few minutes, the air was replaced by BSS.
- Donor DM roll with BSS was sucked into a custom-made injector system (made from IOL cartridge) attached to a 2-ml disposable syringe.
- The donor DM-roll was injected into the anterior chamber through the limbal tunnel. Utmost care was taken that the tip of the cartridge should be well within the anterior chamber while injecting.
- The tunnel was immediately secured with a 10-0 nylon suture, to prevent DM-roll extrusion out of AC during donor manipulation.
- DM-roll was manipulated carefully by repeated tapping over the cornea in the shallow anterior chamber and injecting BSS intermittently till the DM-roll opened with its edges facing upwards towards the corneal stroma. It was evident by observing the correct orientation of ‘S’ mark.
- Intermittent further tapping in the centre of the cornea and simultaneous shallowing of the AC by further draining of the BSS slowly *via *the side port incisions helped complete unscrolling of the DM-roll.
- Air bubble was then injected carefully underneath the centre of the donor DM graft with a 30G cannula to attach the DM-endothelium complex to the recipient stroma.
- The AC was filled further with more air (just complete air-fill, not to overfill) to achieve sufficient air-tamponade effect.
- In some patients with epithelial debridement, a Bandage Contact Lens (BCL) was put at the end of surgery to give comfort to the patient.
- The patient was shifted to the recovery room and was asked to lie down in the supine position for at least one hour.
- After one hour, the patient was examined under slit lamp to check aqueous level and air bubble status in AC. If the fluid level was above the inferior PI, the patient was asked to sit intermittently. If the fluid level was not visible, little air was released *via *one of the side-ports and instantaneously fluid level was formed and remains above the PI.
- As a hospital policy, all patients were kept overnight in the hospital and next morning they were discharged after slit lamp examination.

### 2.3 Postoperative care and Follow up

Postoperatively, all patients received tapering doses of prednisolone acetate eye drop (initially 6 times/day for 2 weeks, then 4 times/day for 2 months, then 3 times, 2 times and at the end of six month once daily to continue) and moxifloxacin eye drop - 4 times daily for 2 weeks and lubricant eye drops - 4 times daily for 6 months. BCL was removed after 7 days.

All patients were examined after one week, one month, three months and six months after the surgery. During each visit, BSCVA was measured using the Snellen chart. Detailed Slit lamp examination was performed to check the graft attachment and IOP measurement was checked with GAT or NCT. Anterior Segment Optical Coherence Tomography (ASOCT) (OPTOVUE, Optovue Inc. Fremont, CA, USA) was also done routinely in all eyes. Clinical non-contact specular microscopy (CELLCHEK SL, Konan Medical Inc., Hyogo, Japan) was done at one month, 3 months and 6 months by a trained technician, who was masked about the procedure. Endothelial Cell Density (ECD) was considered after taking an average of three measurements of the central corneal specular images.

The data was collected and tabulated using Microsoft Excel (Office 2013, Microsoft, USA). The results of continuous measurements were presented as mean ± standard deviation (mean ± SD) and the range; and the results of categorical measurements were presented as number (percentage, %). Paired *t*-test (two-tailed, dependent) and *Chi-square* test were used to find the significance of different study parameters. A *p*-value of <0.05 was considered statistically significant

## 3 RESULTS

**Fig. (1):**
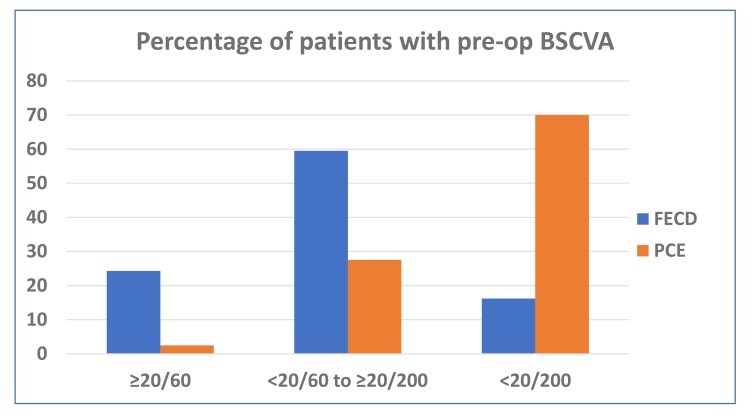
Percentage of patients with preoperative Best Corrected Spectacle Visual Acuity (BSCVA) between Fuchs’ Endothelial Corneal Dystrophy (FECD) and Pseudophakic Corneal Edema (PCE) [only considering the patients without any comorbidity: Total number = 77; FECD = 37 and PCE = 40] *(Chi sq test: p value =0.000055)*.

100 consecutive eyes of 95 patients were included in this study. There were 56 males and 39 females with a mean age of 62.3 ± 10.4 years (range: 29 to 80 years). All patients had clinically significant corneal stromal edema, microcystic epithelial edema or frank bullous keratopathy. 43 (43%) eyes were phakic and 56 (56%) were pseudophakic. 38 (38%) eyes had moderate Fuchs′ endothelial corneal dystrophy with various grades of cataract. In all phakic eyes - DMEK was combined with PE-PCIOL simultaneously (DMEK-triple). 53 (53%) eyes had pseudophakic corneal edema or bullous keratopathy. Preoperatively, in 56% of eyes, the BSCVA in the affected eye were between Light Perception (LP) to <20/200 (Table **1**). Excluding patients with vision affecting comorbidities, the PCE/PBK patients presented with significantly worse BSCVA than the FECD group (Chi sq test: *p*=0.000055) (Fig. **1**).

105 donor corneas were used in this series for 100 DMEK eyes. The donor age was in between 42 to 89 years (65.9 ± 8.3 years). In 3 cases, the Donor DMs were damaged during DM graft preparation and two DM-roll was damage during unscrolling. The mean DM-roll preparation time was 7.5 ± 2.8 min (range: 3.5 - 12 min). Time was calculated after watching the video records – from the placement of donor CS button on Teflon block to shifting of the DM-roll in BSS filled petri dish. Peeling time between MKM and CSM preserved cornea was not statistically significant (*p*=0.6646) (Table **2**).

**Fig. (2):**
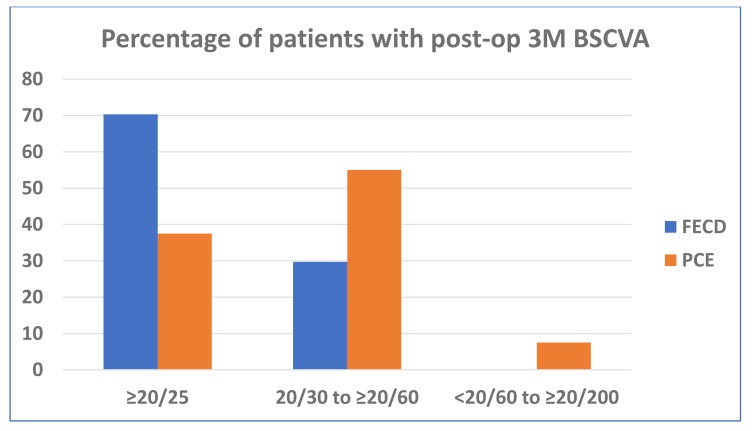
Percentage of patients with postoperative best corrected spectacle visual acuity (BSCVA) after 3 months between Fuchs’ Endothelial Corneal Dystrophy (FECD) and pseudophakic corneal edema (PCE) [only considering the patients without any comorbidity: Total number = 77; FECD = 37 and PCE = 40] *(Chi sq test: p value =0.0086)*.

The results in this DMEK series were highly encouraging in different types of endothelial diseases. After three months 57 (57.6%) eyes regained 20/25 or better vision with very small refractive corrections. The post-operative BSCVA in FECD patients was significantly better than the PCE/PBK patients, not considering eyes with visual comorbidities. (Fig. **2**). The details of the operative results are shown in Table **3** 19 (19%) eyes had co-morbid conditions, for which visual outcomes were unsatisfactory. During donor manipulation, the edges of two donor DM-roll were stuck and unscrolling were difficult. Eventually, they were damaged, and immediately changed with new donor DM-roll and surgery completed. The mean Endothelial Cell Loss (ECL) after 3 months was 26.92 ± 13.40% (range: 4.90% – 66.6%). The commonest complications observed in this series was DM detachment in various form in 9 (9%) eyes and rebubbling required in 4 eyes. In peripheral DM detachment eyes, 5 DM-graft attached spontaneously with time. Iatrogenic primary donor failure happened only in one eye (1%) and re-DMEK was done after 3 weeks.

## 4 DISCUSSION

Descemet membrane endothelial keratoplasty or DMEK is an emerging and more advanced form of endothelial keratoplasty alternative DSEK/DSAEK for corneal endothelial diseases. In this study, we evaluated the surgical and visual outcomes of 100 consecutive DMEK cases who underwent operation in our tertiary care hospital. All the surgeries were performed by a single surgeon using same standardize ‘no touch’ technique and DM graft preparation was done by the surgeon himself in the operating room before each case.

Our study showed that overall 27.3% of eyes achieved an UCVA of ≥20/25 and 57.6% eyes achieved BSCVA of 20/25 or more after 3 months. If we exclude 19 eyes with different co-morbidities, these figures would have been 33.7% and 71.2% respectively. Previous studies in different series also similar agreement that up to 75% DMEK eyes may achieve BSCVA of 20/25 or more [11-16]. The visual outcomes in DMEK are better than that after DSEK/DSAEK and approximately 35% of the eyes may reach a BSCVA of 20/25 or more at 6 months postoperatively [8, 16]. We have a different patient population in this series – the majority of our patients were pseudophakic corneal edema or frank bullous keratopathy and in contrast Fuchs’ dystrophy cases were less. Furthermore, the presenting preoperative vision in 56% of eyes was between Light Perception (LP) to <20/200 which was also much less as compared to western reports [5, 17].

The mean ECD was 2123 ± 438 cells/mm^2^ (range 976 to 3208 cells/ mm^2^) at 3 months postoperatively in this series. The mean endothelial cell loss (ECL) at 3 months was 26.92 ± 13.40% in our study, which is similar to other studies on DMEK or DSEK/DSAEK in the literature [7, 11, 15, 18, 19]. In a small series, Bhandari *et al*. from India showed that ECL was only 24% after 6 months postoperatively which is less, and they explained that the preparation by the surgeon was important and manipulation was less traumatic [10]. In this series, we have also observed that the early post-mortem use of donor tissue with surgeon’s preparation of donor during surgery and no-touch technique during donor manipulation - all are probably important to reduce the endothelial cell loss during surgery.

Another important observation in our series is that there were less number of complications. We had only 9% DM detachment of several types and rebubbling required in 4% eyes to re-attached the grafts. Iatrogenic primary graft failure was only 1% which was also less than other studies [5, 6, 15, 17]. This is probably due to multiple factors – like, experienced DSEK surgeon performed more than 1000 procedures with past knowledge of avoiding complications; standardization of DMEK technique over time, and late starter with knowledge gathered from the literature about DMEK complications and their prevention. Philips *et al*. recently published their comparable results - that with experienced DSAEK surgeon, the transition to DMEK learning was not that steep with minimum complications [20].

During surgery, in two cases, donor unscrolling was not possible because the edges of the donor DM-roll was stuck as if they were glued with fibrin. It happened in an early part of DMEK transition. In both the cases, DM-roll was removed, through AC wash was given with BSS and followed by air tamponade. After few minutes, the air was replaced by BSS, and then a second DM-roll injected and unscrolled. In some eyes, we observed small bleeding occurred from the inferior PI site immediately after iridectomy. Even after AC wash with BSS, very little amount of oozing continues. So, we recommend, after inferior PI, immediate anterior chamber wash with BSS, followed by air tamponade. This is to stop this slow oozing of blood or fibrin from PI site and DM-roll unscrolling will be uneventful.

There are a few important different highlighting points and observations in this study. Firstly, we used both MK medium- and Cornisol- preserved corneas for the harvesting of donor DM graft. The costs of these media are less than Optisol-GS which are suitable for the developing countries. Bhandari *et al* also used Cornisol-preserved donor corneas for DM graft preparation [10]. Published *in vitro* study showed that there is no difference in endothelial viability between the donor corneas stored in Cornisol and Optisol-GS media up to 14 days of storage [9]. Secondly, our patient populations with endothelial diseases were different: we have more PCE/PBK than FECD. Moreover, our patients presented late with presenting visual acuity much less than Western population data. Thirdly, the majority of the Indian eyes are different from the eyes of the Western world. Like other Asian people, Indians have brown or dark brown iris in the majority of the eyes and a relatively shallow anterior chamber [21-23]. In these eyes, DM graft manipulation in the anterior chamber is difficult since DM-roll does not take its shape easily in presence of shallow anterior chamber and the edges of DM-roll are not easily visible in darker iris background. That is why ‘S’ mark (or other mark) on DM-side during graft preparation is very helpful for right orientation of DM unscrolling and its attachment. It also saves time. In this series, all DM-roll were unscrolled in right orientation before air injection.

## CONCLUSION

In conclusion, Descemet membrane endothelial keratoplasty is a safe and effective procedure in Indian eyes with endothelial diseases with encouraging surgical and better visual outcomes. Complications are less and endothelial cell loss is acceptable. However, the present study has limitation about the short follow-up period. A longer follow up is necessary to comment on the long-term endothelial cell loss and rejection incidence in case of DMEK among the Indian population.

## Figures and Tables

**Table 1:** Patients’ profile and preoperative data (95 patients; 100 eyes).

**Age** (Year; Mean and range)	62.3 ± 10.4	29 -80
**Male: Female**	56: 39	
**Preoperative BSCVA**		
LP to <20/200	56	56%
20/200 to 20/60	39	39%
<20/60 to 20/40	5	
** Indications**		
PCE or PBK	52	52%
FECD with cataract	38	38%
Post PK failed graft	4	4%
HSV endotheliitis-induced corneal edema	2	2%
CMV endotheliitis-induced corneal edema	1	1%
Failed DMEK (IPGF)	1	1%
ICE syndrome	1	1%
Aphakic bullous keratopathy (ABK)	1	1%
**Lens status**		
Pseudophakic	55	55%
Phakic	43	43%
Aphakic	1	1%
**LP** – Light Perception; **PCE** – Pseudophakic Corneal Edema; **PBK** – Pseudohakic Bullous Keratopathy; **FECD** – Fuchs’ Endothelial Corneal Dystrophy; **PK** – Penetrating Keratoplasty; **HSV** – Herpes Simplex Virus; **CMV** – Cytomegalovirus; **IPGF** – Iatrogenic Primary Graft Failure; **ICE** – Iridocorneo Endothelial		

**Table 2:** Donor characteristics and DM-roll preparation.

	**MKM preserved cornea**	**CSM preserved cornea**
Number of cornea	51*	54**
Age of donor (Year; Mean; range)	65.2 ± 9.9 (42 - 82)	67.1 ± 6.4 (53 - 89)
Death to media time (Min; mean)	247 ± 77	242 ± 67
Media to surgery time (Hour: mean)	107 ± 40	39 ± 16
DM-roll preparation time(Min: Mean; range)	7.4 ± 2.2 (4.5 - 12)	7.6 ± 3.3 (3.5 - 17) #
Endothelial cell density (cell/mm^2^:Mean; Range)	2900 ± 243 (2500- 3717)	2912 ± 219 (2521-3636)
**MKM** – McCarey Kaufman Medium; **CSM** – Cornisol Medium; * 2 donor damaged during DM peeling; ** 1 donor damaged during DM peeling; **# *p*-value** = 0.6646		

**Table 3:** Overall visual and surgical outcomes (*n*= 100).

**Visual acuity, ECD, ECL and complications**	
**Postoperative ** **UCVA (*n*=99)**	–
**** ▪ 20/25 or better	27 (27.3%
▪ 20/30 to 20/60	55 (55.6%)
▪ <20/60 to 20/200	18 (18.2%)
▪ <20/200	09 (9.1%)
**Postoperative BSCVA (*n*=99)**	–
▪ 20/25 or better	57 (57.6%)
▪ 20/30 to 20/60	27 (31.4%)
▪ <20/60 to 20/200	10 (10.2%)
▪ <20/200	05 (5.1%)
** Endothelial cell density – Donor**	–
[Cells/sq mm; Mean ± SD (range)]	2891 ± 227 (2500 – 3717)
**Endothelial cell density – after 3 months**	–
[Cells/mm^2^; Mean ± SD (range)]	2123 ± 438 (976 – 3208)
**Endothelial cell loss (ECL)**	–
Percentage; Mean ± SD (range)	26.92 ± 13.40 (4.90% – 66.6%)
**Complications - n; (%)**	–
▪ Graft damaged during preparation	3 (3%)
▪ Graft damaged during unscrolling	2 (2%)
▪ Pupillary block	2 (2%)
▪ Detachment of DM graft	–
*Total*	1 (1%)
*Partial central*	2 (2%)
*Partial peripheral*	6 (6%)
▪ Iatrogenic primary donor failure	1 (1%)
▪ Secondary glaucoma	7 (7%)
▪ HSV recurrence	1 (1%)
▪ Infection/rejection	Nil 19
▪ Comorbid other ocular conditions	(19%)
**Reoperations required:**	–
▪ Rebubbling	4 (4%)
▪ Re DMEK	1 (1%)
**UCVA:** Uncorrected Visual Acuity; **BSCVA:** Best Spectacle Corrected Visual Acuity; **DM:** Descemet Membrane; **HSV:** Herpes Simplex Virus	
